# Novel Minimally Invasive Mastoid Sling for a Nonsurgical Neck Lift: A Combination of Liposuction, Thermal Energy, and Suture Suspension for Neck Rejuvenation

**DOI:** 10.1093/asjof/ojag065

**Published:** 2026-04-11

**Authors:** R Brannon Claytor, Patricia M Fuentes, Grace Tolan, Lauren Lowe

## Abstract

**Background:**

Neck aging manifests as blunting of the cervicomental angle (CMA), loss of jawline definition, vertical platysmal banding, submental fat accumulation, and redundant skin. While open neck rejuvenation techniques produce dramatic and durable results, they require extensive dissection, which can have prolonged recovery times. Today, many patients are seeking less invasive options with faster recovery times and optimal results. Previous attempts in our field to conduct a minimally invasive neck lift may lack sufficient deep anchoring to provide long-term support, particularly in the submandibular region.

**Objectives:**

Therefore, the objective of this paper is to introduce the minimally invasive mastoid sling (MIMS) neck lift technique, a minimally invasive neck lift technique that combined liposuction, thermal energy, and permanent suture suspension for improved neck aesthetics.

**Methods:**

All patients who underwent the MIMS neck lift at a single surgical center (2020-2024) were included. The procedure was conducted under tumescent local anesthesia in a QUAD-A surgical facility accredited by the American Association for Accreditation of Ambulatory Surgery Facilities (AAAA SF). Demographics, operative variables, and postoperative outcomes were collected. Descriptive analysis was completed.

**Results:**

A total of 73 patients were included, of which 67 (91.7%) were female. The mean age was 48.7 years (range: 22-82) and the mean BMI was 25.44 kg/m^2^ (range: 19-38). The overall complication rate was 5.4% (n = 4), with seroma occurring in 3 patients (4.1%) and neuropraxia in 1 patient (1.4%). There were no long-term sequelae, and no revision surgeries were performed within a year.

**Conclusions:**

Although open platysmaplasty remains the definitive treatment for neck aesthetics, the trifecta of liposuction, thermal energy, and permanent suture suspension anchored on the mastoid sling provides an effective, minimally invasive alternative with a low complication profile.

**Level of Evidence: 4 (Therapeutic):**

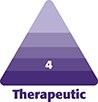

The neck is often the first facial feature to show signs of aging, with blunting of the cervicomental angle (CMA), loss of a defined jawline, platysmal banding, submental fat accumulation, and redundant skin.^1^ These changes can significantly affect overall facial aesthetics, and their correction has long been the focus of neck rejuvenation surgery. From Ellenbogen et al to Halani et al, both described the ideal neck as a sharp CMA of 90 to 120° and a gonial angle of 120 to 140°.^2,3^ These are the current gold standard benchmarks for neck and jawline aesthetics.

Historically, rejuvenation techniques evolved from superficial skin tightening to deeper, structural procedures targeting the platysma, submental and subplatysmal fat, and submandibular glands.^4,5^ Feldman's corset platysmaplasty, introduced in 1988, combined midline and lateral platysmal plication with resection of submental fat, anterior digastric muscles, and submandibular glands to restore cervical definition.^6^ Shortly thereafter, Giampapa and DiBernardo developed the suture-suspension neck lift, later refined by Giampapa in 1995 with an interlocking mastoid-to-mastoid suspension suture. Other contributions, such as Connell's platysma flap anchored to the mastoid fascia, Guerrero-Santos's 2-flap platysma sling, and Baker's reinforced mastoid anchoring, further advanced the field, often in combination with facelift techniques to achieve durable results—though at the expense of greater dissection, longer recovery, and higher complication risks.^4,7,8^

In recent years, patient demand has shifted toward less invasive procedures with shorter recovery and in-office feasibility, leading to the development of a wide range of nonsurgical alternatives to deliver improved jawline definition.^9-11^ However, the unique anatomy and dynamic forces of the neck, particularly mimetic activity and fat distribution, limit the effectiveness of approaches that rely solely on skin traction or superficial support.^12^

There are options for minimally invasive techniques that can produce sharper jawlines. One such technique is the percutaneous platysmaplasty, introduced by Mueller, which uses a transcutaneous light-emitting diode (LED)-guided system to pass a permanent suture matrix along the platysma, anchored to neck-retaining ligaments at the mandibular border.^13^ This approach avoids large incisions and provides cervical support, but results may be modest and less durable, with recurrent laxity possible over time or with mandibular motion.^14^ Other options include energy-based skin tightening and injectable treatments, however, the longevity of these treatments are variable.^15,16^

The objective of this paper is to introduce the minimally invasive mastoid sling (MIMS) neck lift technique that combines liposuction and laser thermal energy with a dual-suture system to anchor a permanent suture to the dissected mastoid fascia and a second superficial absorbable suture that targets skin laxity.

## METHODS

### Study Design

This retrospective case series included all patients who underwent the minimally invasive mastoid sling (MIMS) neck lift performed by the primary surgeon between December 2020 and August 2024. IRB exemption (E-24-5420) by the Main Line Health was granted on September 16, 2025. The patients included provided informed consent, and several patients signed media release forms for their photographs to be used. A descriptive analysis of patient demographics was performed using SPSS v30.0 (IBM, Armonk, NY).

### Surgical Technique

Video 1, available online at www.asjopenforum.com, summarizes all surgical steps. The primary surgeon performs the minimally invasive mastoid sling neck lift under tumescent local anesthesia at a Class A American Association for Accreditation of Ambulatory Surgery Facilities (AAAA SF) (QUAD-A)-certified surgical facility. The neck is tumesced with approximately 250 cc solution of normal saline infused with lidocaine and epinephrine. First, submental fat was removed with power-assisted liposuction in the preplatysmal plane. Followed by laser thermal heating using the SmartLipo (Cynosure, Westford, MA) performed into the subdermis and subcutaneous layers delivering thermal energy to the soft tissue. Liposuction and laser thermal heating are completed prior to suture suspension.

**Figure 1.**
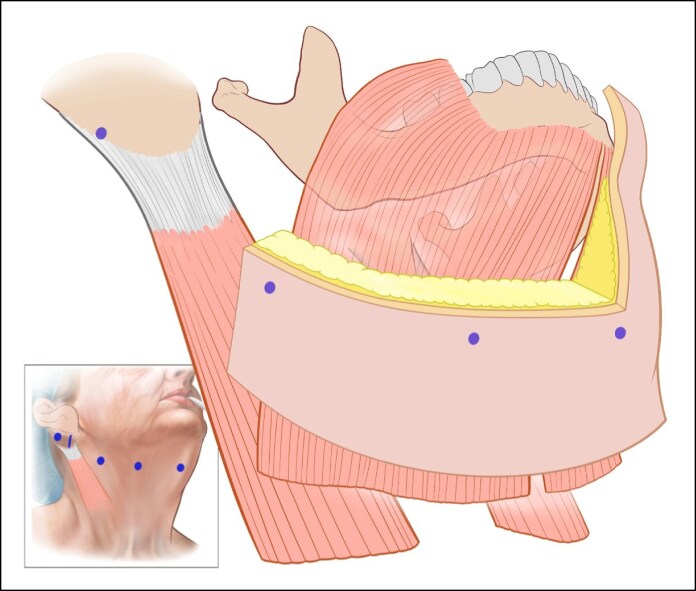
Puncture sites are created centrally 1 cm superior to the body of the hyoid bone, directly over the submandibular gland, midportion of the body of the mandible, and inferior to the angle of the mandible.

Anticipated puncture sites are made in these areas: (1) centrally 1 cm superior to the body of the hyoid bone, lateral to this, (2) directly over the submandibular gland, (3) midportion of the body of the mandible, and (4) inferior to the angle of the mandible (Figure 1).

In the postauricular region, directly over the mastoid process, an oblique 1 cm incision is made, extending through the skin and the mastoid fascia, superficial to the periosteum. A superiorly oblique tunnel, measuring 1 cm by 2 cm, is created to form the mastoid fascial tunnel, with communication made through the skin at the distal aspect of the tunnel. A separate subcutaneous tunnel is created directly overlying the mastoid fascia tunnel. This is also connected through the skin.

With a 4-0 Ethibond (Ethicon, Inc., Raritan, NJ), this allows the suture to be placed deep to the mastoid fascia along the mastoid bone and then brought through the skin and reintroduced into the subcutaneous tunnel. This creates a cerclage grasping attachment to the mastoid fascia, providing a strong anchor that serves as the mainstay of this innovative technique.

**Figure 2.**
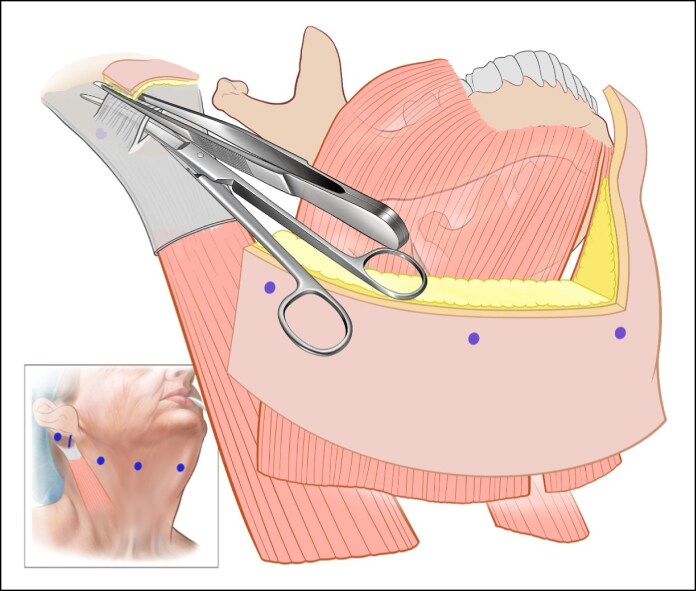
To develop the mastoid sling, an oblique incision is made directly overlying the mastoid bone. A superficial tunnel is made between the skin and mastoid fascia. A second pocket is made deep to the mastoid fascia.

**Figure 3.**
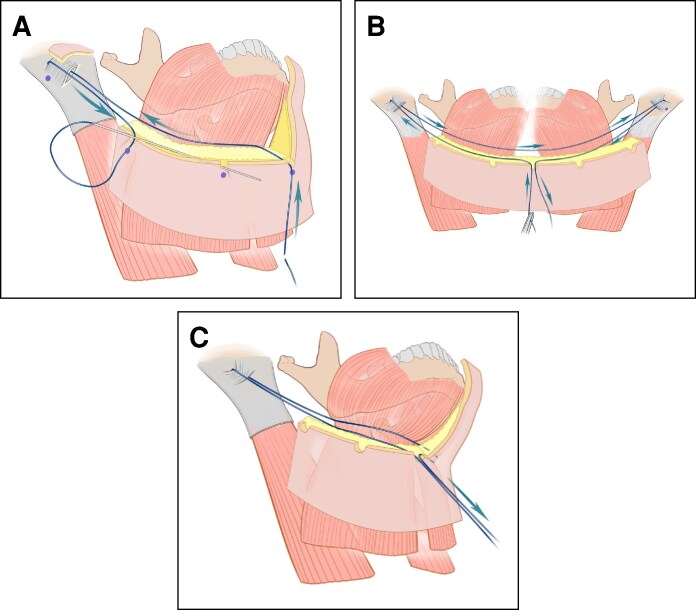
(A) A 4-0 Ethibond suture is deployed at the neck midline. The suture is threaded deep to the dermis and traversed from the neck midline to the mastoid fascia. (B) The suture is passed through the tunnel deep to the mastoid fascia and then back through the superficial tunnel to fully envelope the mastoid bone. (C) The suture is delivered through the skin, passed inferiorly to the neck midline to reach the contralateral side, and similarly passed around the mastoid bone.

The suture is percutaneously passed through subcutaneous tunnels across the anterior neck to the contralateral side and similarly passed around the mastoid fascia sling and then brought back centrally in the neck and temporarily secured with a hemostat (Figures 2, 3).

A second suture, which is a 4-0 Monocryl (Ethicon, Inc., Raritan, NJ), is introduced through a separate puncture site directly over the submandibular gland on the left side. This suture is passed through a similar path as the 4-0 Ethibond; however, the 4-0 Monocryl is placed into the deep dermis, whereas the 4-0 Ethibond is placed deep to the dermis. It is similarly passed around the mastoid sling on each side and brought out through a percutaneous hole directly over the left submandibular gland.

The operating room table is adjusted to flex the patient's neck to a 30° angle. The 4-0 Ethibond is tied with sufficient force to hold the suture in place while manipulating the tissue to ensure no puckering of the skin is seen. Then, the 4-0 Monocryl suture is tied tightly to create significant skin puckering.

Lastly, a subsequent hemostatic net suture is placed from the posterior region across the jawline and the hyoid bone to the contralateral side. The puncture sutures are covered by the hemostatic net suture. No drains are placed. Sutures are left in place for 3 days. Patients remain wrapped for 1 week.

### Cadaver Dissection

In August 2025, a fresh cadaver dissection was conducted at the anatomy lab of the Miami Anatomical Research Center (MARC) to evaluate the efficacy and tensile strength of the MIMS neck lift technique. A 4-0 Ethibond permanent suture was started at the neck midline, superior to the hyoid bone. The suture was threaded deep under the dermis to create a tunnel around the mastoid fascia in the retroauricular region. The suture was passed under the mastoid tunnel, through the skin and back, securely anchoring it around the mastoid fascia to create a sling. Then, a 4-0 Monocryl suture was placed superficially just below the epidermis. The Ethibond, followed by the Monocryl suture, was tied tightly (Video 2, available online at https://doi.org/10.1093/asjof/ojag065).

## RESULTS

A total of 73 patients underwent the MIMS neck lift. The mean age was 48.7 years (range: 22-82), and the mean BMI was 25.44 kg/m^2^ (range: 19-38). Patients selected for this procedure typically presented with excess submental fat and modest excess neck skin (Table 1).

**Table 1.** Demographic and Procedural Characteristics

Characteristic	Value
Total patients, *n*	73^a^
Age, years	
Mean ± SD	48.78 ± 12.89
Range	22-82
Sex, *n*	
Female	67
Male	6
BMI, kg/m^2^	
Mean ± SD	25.44 ± 3.77
Range	19-38
Operative time, minutes	
Mean ± SD	150.57 ± 74.78
Range	57-415
Amount of tumescent, mL	
Mean ± SD	293.89 ± 134.17
Range	40-560
Amount of local anesthetic, mL	
Mean ± SD	33.48 ± 10.94
Range	15-60
Additional procedures	62 (80.5%)

SD, standard deviation.

^a^Indicates all patients who underwent the minimally invasive mastoid sling (MIMS) technique.

Table 2 summarizes all postoperative outcomes. In this cohort, the most common complication was seroma which developed in 3 patients, all of whom were managed with in-office aspiration, without any sequelae. Seroma volumes ranged from 1 to 3 cc and occurred within days of surgery: Patient 1 developed a 3 mL seroma on postoperative Day 8; Patient 2 developed a 2 mL seroma on postoperative Day 1; and Patient 3 developed a 1 mL seroma on postoperative Day 11. One patient experienced neuropraxia of the right marginal mandibular nerve that presented on postoperative Day 5 and resolved spontaneously within 2 weeks without residual deficit.

**Table 2.** Post-procedure Outcomes

Characteristic	Value
Excessive skin puckering, *n* (%)	13 (16.9%)
Microneedling^a^	6 (8.2%)
Microneedling + radiofrequency^a^	3 (4.1%)
Puckering resolution, days	
Mean ± SD	24.75 ± 8.79
Range	16 - 42
Total complications, *n* (%)	4 (5.4%)
Seroma	3 (4.1%)
Neuropraxia	1 (1.4%)
Hematoma	0
Skin necrosis	0

^a^Indicates patients with excessive skin puckering who underwent additional microneedling with or without radiofrequency.

**Figure 4.**
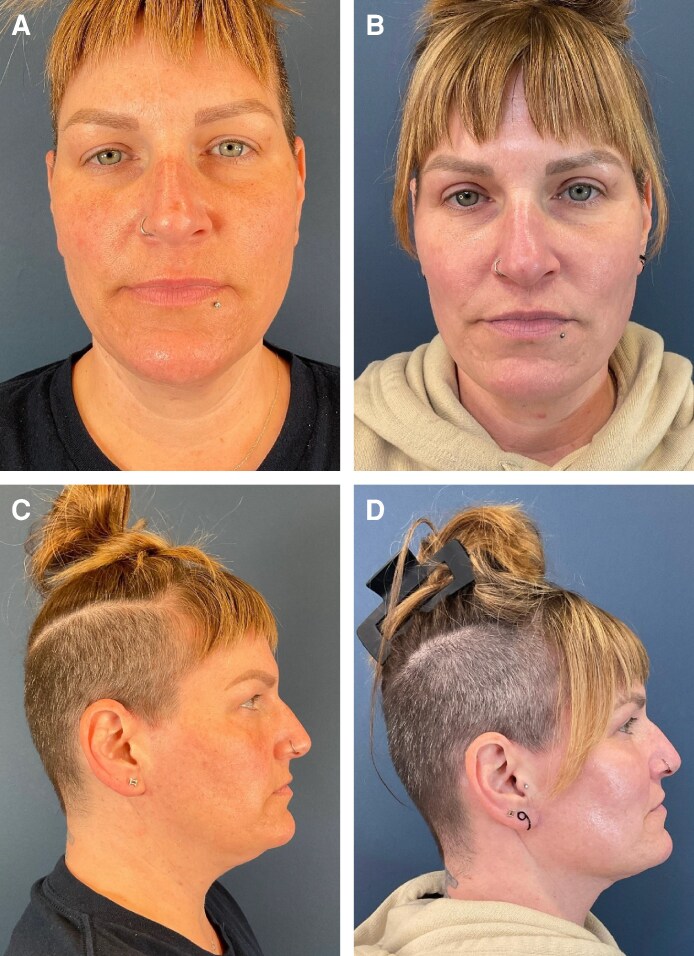
A 45-year-old female patient 1 year after the minimally invasive mastoid sling (MIMS) technique. (A and C) Preoperative. (B and D) One-year follow-up.

**Figure 5.**
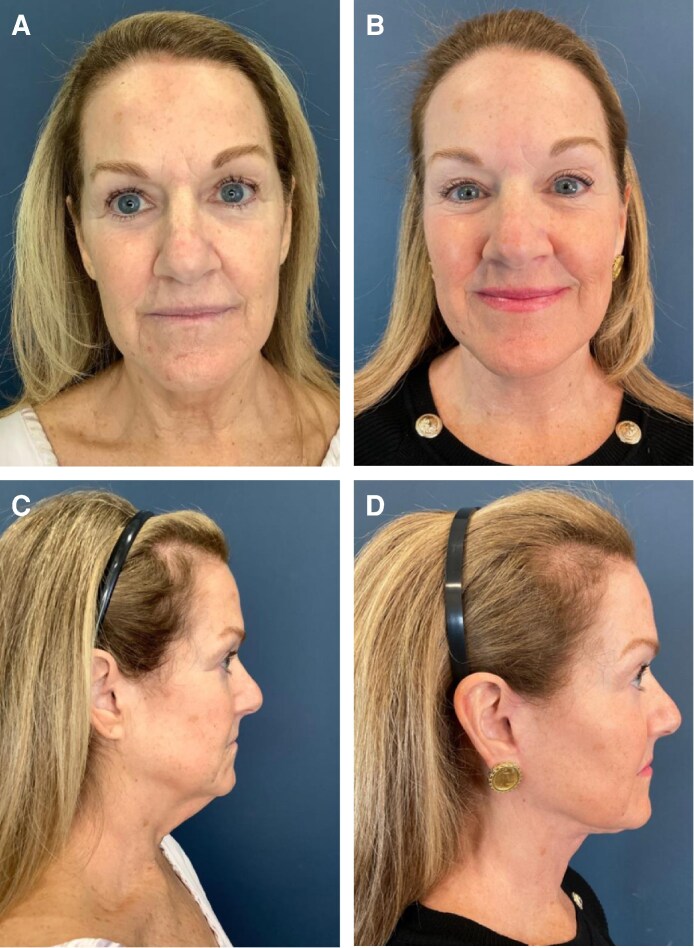
A 67-year-old female patient 1 year after the minimally invasive mastoid sling (MIMS) technique. (A and C) Preoperative. (B and D) One-year follow-up.

**Figure 6.**
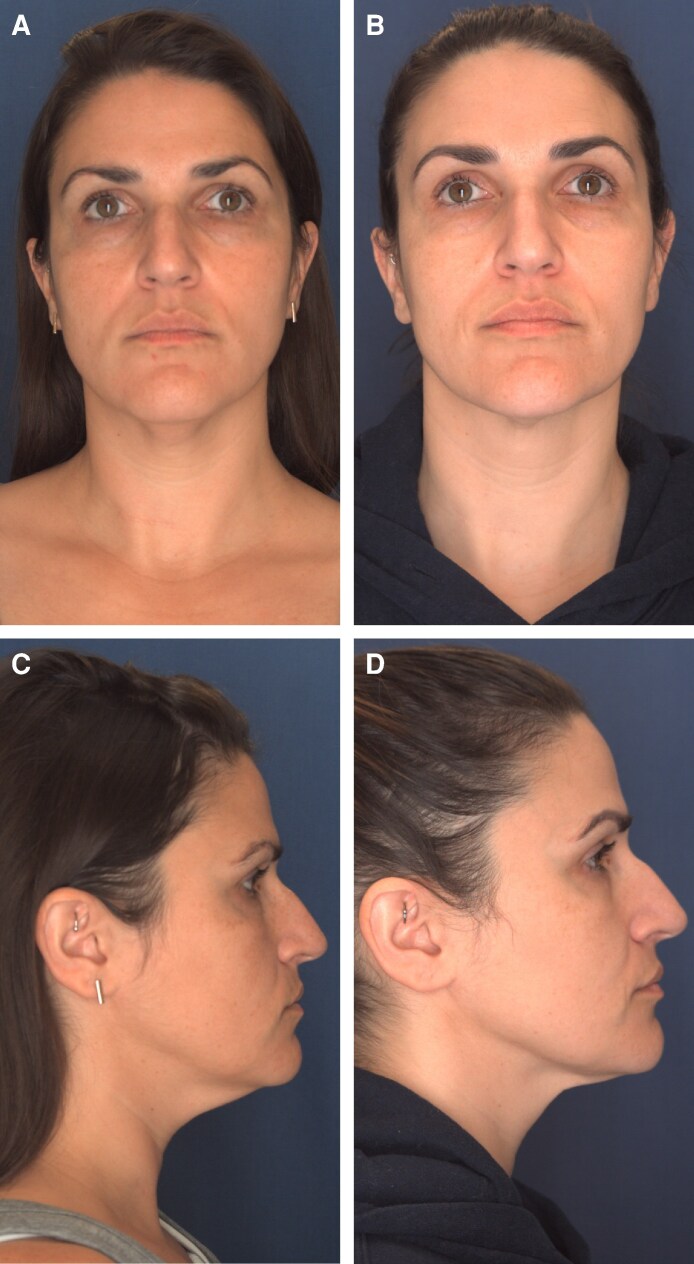
A 40-year-old female patient 1 year after the minimally invasive mastoid sling (MIMS) technique. (A and C) Preoperative. (B and D) Two-year follow-up.

A total of 13 patients (16.9%) underwent additional treatments for excessive skin puckering, of whom 6 (8.2%) were treated with microneedling alone and 3 (4.1%) with microneedling plus radiofrequency. Overall, the mean time to resolution of skin puckering was 24.75 days (range: 16-42). Clinical outcomes at 1- and 2-year follow-ups are demonstrated in Figures 4 to 6. The dynamic neck video is available in Video 3, accessible online at www.asjopenforum.com.

## DISCUSSION

With the growing interest in minimally invasive procedures that provide durable and effective aesthetic results, surgeons have sought to address the cosmetically vexing problem of the aging, sagging neck through less invasive approaches. Therefore, this paper describes the MIMS neck lift, a modified dual-suture suspension technique that combines suture suspension with liposuction and thermal injury designed to restore cervical definition and youthfulness.

Open platysmaplasty remains the gold standard treatment for cervical rejuvenation in patients with advanced neck aging, including blunting of the cervicomental and gonial angles, dynamic platysmal banding, and poor skin elasticity.^1,3,17^ However, many patients who present with less severe soft tissue atrophy, submental fullness, platysmal muscle banding, and submandibular gland ptosis do not require the extensive dissection of a traditional open neck lift technique to achieve a youthful and defined cervicomental and gonial angles.^10,12^ With the growing preference for minimally invasive treatments, patients desire techniques that offer decreased downtime while achieving highly desirable outcomes.^18,19^ This shift in the cosmetic market for minimally invasive rejuvenation has motivated surgeons to develop minimally invasive or nonsurgical techniques that provide effective and durable results.^20^ Patient selection remains critical when deciding between a minimally invasive and an open neck lift approach based on the degree of laxity, jowling, and banding, guiding the surgeon in determining when minimally invasive approaches may be sufficient to achieve aesthetic outcomes.^21,22^ The MIMS neck lift is most suitable for patients with early-to-minimal neck skin laxity and excess submental fat, who do not require extensive dissection of the neck to achieve the desired aesthetic outcome.

Giampapa introduced the interlocking suture suspension for minimally invasive neck lifting, which deploys a single, long, permanent interlocking suture placed in the subcutaneous plane to create a supportive ligament under the mandible.^1,10^ The difference between Giampapa's technique and the MIMS is the strong mastoid fascia anchor, which provides a durable and reliable traction point to transmit elevating forces over the submandibular glands and underneath the jaw. The secondary absorbable suture as well as the hemostatic net suture adds to the neck contouring and sculpting with tissue manipulation that maximizes lifted and repositioned tissue to adhere to the new contour through temporary suture adhesion. Moreover, Mueller's percutaneous platysmaplasty uses a light-guided system that introduces a continuous suture matrix along the platysma and anchors it to cervical retaining ligaments, creating a trampoline-like support framework.^13^ More recently, Kaplan et al described a single-incision approach performed entirely through a central cervicomental incision, thereby eliminating the need for postauricular access while achieving a minimally invasive neck lift.^11^ These innovations reflect a clear trend toward minimally invasive procedures that minimize scarring and reduce downtime and complication risk while still providing meaningful rejuvenation of the cervical contour.

The primary contribution of the MIMS technique is the dual-suture modality of the permanent braided suture placed in a deep layer to contour the neck and jawline based on a strong anchor support of the mastoid fascia and centrally tied knot. Braided sutures have inherently soft tissue characteristics, which make them less palpable and less noticeable at the suture line. The second is the deployment of a 4-0 Monocryl suture in the deep dermis. Monocryl, also known as poliglecaprone 25 suture, causes a mild dermal reaction during would healing, which has been characterized as minimal inflammation with infiltration of mononuclear cells and mild fibrin synthesis.^23,24^ Employing this absorbable second suture temporarily redistributes the loose skin to the platysmal and fascial layers, which may promote fibrin-mediated adhesion of the skin to the new neck contour. As these sutures dissolve, the undulating and irregular skin folds relax, leaving a soft, smooth contour that is adherent to the new neck contour, therefore achieving an improved CMA.

Building upon these principles, the MIMS neck lift offers an alternative approach by also combining soft-tissue debulking with power-assisted liposuction and thermal energy skin tightening with the permanent suspension. The technique employs both a permanent and an absorbable suture through subdermal tunnels. A critical initial step is thorough power-assisted liposuction of the preplatysmal plane, which not only evacuates excess adipose tissue but also assists in creating subdermal channels that facilitate more precise suture placement.

Isolated cervicomental liposuction is a well-established option for patients with favorable skin elasticity and minimal platysmal banding; however, when used alone, it primarily targets preplatysmal fat without significant skin-tightening benefits.^25^ Combining liposuction with thermal energy induces lipolysis to liquefy adipocytes, targeting preplatysmal fat removal while also delivering a skin-tightening effect through neocollagenesis and adipolysis.^26^ This combination treatment promotes collagen production and simultaneously cauterizes small blood vessels, which decreases bleeding and swelling. Badin et al and Ichikawa et al showed in their histological studies that laser lipolysis can rupture fibrous septa, freeing retracted skin and remodel dermal collagen, resulting in clinical skin tightening. Studies have shown that inducing dermal temperature between 60 and 70°C is associated with collagen contraction and neocollagenesis.^27,28^ By integrating power-assisted liposuction and laser thermal energy, the MIMS technique reduces preplatysmal fat and creates a subdermal plane through which thermal energy can be delivered directly to the dermis, inducing neocollagenesis that results in clinically visible skin tightening. The importance of comprehensive power-assisted liposuction to evacuate the fat from the preplatysmal plane as well as allowing for heat energy devices to aid in the shrinking of loose dermal elements is an essential step for preparing the neck for suture deployment.

In this cohort, the hemostatic net was maintained for 4 days postoperatively to solidify these new skin-to-platysma connections through a quilting pattern. By evenly compressing the tissues captured within the net, these sutures also help minimize dead space and promote hemostasis. Pellini et al reported, in a retrospective cohort study of 480 cervicofacial rhytidectomy patients, a reduction in hematoma formation from 5.42% to 0% after implementing a hemostatic net suture.^29^ Moreover, previous studies have demonstrated that the hemostatic net does not compromise skin blood supply and perfusion.^30,31^ This finding is corroborated by our study in which no hematomas or skin necrosis occurred. Beyond hemostasis, suture techniques have been found to influence collagen deposition and wound healing.^32^ Ismail et al sought to demonstrate if a hemostatic net suture induced collagen remodeling or synthesis using a rat model; however, at 20 days, the findings did not detect any significant differences in neocollagenesis, although the authors acknowledged that the small sample size may have limited their ability to detect subtle effects.^33^ Although direct evidence regarding the hemostatic net's impact on collagen synthesis and extracellular matrix remodeling remains limited, this represents an important area for future investigation, particularly in the context of minimally invasive neck rejuvenation techniques.

Microneedling with and without radiofrequency (RF) has been found to be effective in reducing skin folds and puckering. In this study, patients who had skin puckering underwent additional microneedling with or without RF to target these irregularities. A total of 13 patients in this cohort underwent additional treatment for skin puckering. Microneedling alone stimulates dermal remodeling and neocollagenesis by introducing micropunctures into the dermis to improve skin texture and irregularities.^34,35^ Moreover, microneedling with RF combines mechanical injury with thermal energy to augment neocollagenesis, elastogenesis, and dermal extracellular matrix remodeling, providing additional skin tightening.^36,37^ Hwang et al found that microneedling with RF increased the number of nonsenescent fibroblasts in the dermis, thus increasing collagen and elastin synthesis.^38^ These findings suggest that microneedling with or without RF can be used as a postprocedure adjunct to address any residual skin irregularities and optimize minimally invasive neck lifting results.

This study does have limitations worth discussing. It is a single-surgeon series with a small sample size, which limits the generalizability of our findings. Due to its retrospective design, causative conclusions cannot be drawn from the results of this study. Future multicenter, prospective studies are needed to validate the efficacy and reproducibility of the MIMS technique.

## CONCLUSIONS

By combining liposuction, laser thermal energy, and the mastoid sling, this trifecta approach effectively targets early signs of cervical skin laxity. Together, these modalities address excess fat, skin laxity, and structural support, offering a minimally invasive alternative to open platysmaplasty for appropriately selected patients.
